# Supplementary material for: Content-rich biological network constructed by mining PubMed abstracts
Source: BMC Bioinformatics. 2004 Oct 8;5:147. doi: 10.1186/1471-2105-5-147 (PMC528731; doi:10.1186/1471-2105-5-147)
Supplement: Additional File 5 — The original Chilibot query results of the term "long-term potentiation (LTP)" and 22 other terms, limiting the latest references analyzed to the years 1990, 1995, 2000, and 2004. [file 1471-2105-5-147-S5.bz2 › chilibotAdditionalFile5/ltp2000/html/KV4.2.html]

 


**KV4.2** (Input: KV4.2 ) 

---


**Google Searches:** Entire Web  | EDU domain only  | PDF files only

.

**External Links:** OMIM |
LocusLink
| Swissprot
| GeneCards

  
**Maps of KV4.2**

Simple
Complete
graph in
radiant
tree
square
layout.

**New Hypothesis !**

**Synonyms** 

- kv4.2   [PubMed]

**Synopsis**

- These observations indicate that **Kv4.2** is a substrate for ERK in vitro and in vivo, and suggest that ERK may regulate potassium channel function by direct phosphorylation of the pore forming alpha subunit.  J Neurochem, 2000 
  [16]

- These results provide insight into likely sites of regulation of **Kv4.2** by PKA.  J Biol Chem, 2000 
  [14]

- We propose that these functional effects occur as a consequence of the previously reported developmental Kv1.4 to **Kv4.2** Kv4.3 isoform switch.  J Physiol, 1997 
  [10]

- Second, as phosphorylation may regulate channel biophysical properties, differential phosphorylation of **Kv4.2** in the dendrites of pyramidal neurons may confer unique biophysical properties upon particular dendritic input layers.  Learn MemLearn Mem, 1997 
  [10]

- thisinteraction is essential for the generation of appropriate **Kv4.2** current densities.  J Neurosci, 2000 
  [10]

- However, they differ from mammalian preparations in which Shal like **Kv4.2** rather than Shaker like Kv1.4 co localize postsynaptically with glutamate receptors.  J Neurocytol, 1999 
  [10]

- These results provide the necessary functional support for the hypothesis that **Kv4.2** is a major isoform contributing to cardiac ITO, consistent with independent biochemical and molecular evidence that indicates that **Kv4.2** is readily detected in rat myocytes.  Cardiovasc Res, 1997 
  [8]

- Thus **Kv4.2** and Kv4.3 proteins are important subunits of native rat ventricular I to, and PKC appears to reduce this current by affecting the molecular components of the channels mediating I to .  Am J Physiol, 1997 
  [7]

- We observed that exposure to LY294002 or wortmannin, inhibitors of phosphatidylinositol PI 3 kinase, reduced somatic cross sectional area, neurite outgrowth, whole cell capacitance, IA amplitude and density amplitude normalized to membrane area, and immunoreactivity for **Kv4.2** and or Kv4.3 potassium channel subunits likely to be present in the channels carrying IA .  J Neurosci, 1998 
  [4]
